# Embodied Cognition and Alcohol Use Disorder: Frequency of Impairments and Relationship to Neurocognitive Assessments

**DOI:** 10.3390/brainsci15030228

**Published:** 2025-02-22

**Authors:** Morris D. Bell, Andrea J. Weinstein, David Ciosek, Sarah E. Reilly, Yan Wang, Gihyun Yoon

**Affiliations:** 1 Department of Psychiatry, School of Medicine, Yale University, New Haven, CT 06510, USA; 2 VA Connecticut Healthcare System, West Haven, CT 06516, USA

**Keywords:** embodied cognition, alcohol use disorders, cognitive decline, automated test of embodied cognition

## Abstract

**Background**: Embodied cognition is an emerging concept in cognitive science that emphasizes the integral role of perception, action, and bodily experience in shaping human thought and understanding. Recently, a new instrument has been developed called the Automated Test of Embodied Cognition (ATEC), which provides a comprehensive measure of eight domains of embodied cognition. **Method**: An embodied cognition in an alcohol use disorder (AUD) sample (N = 49) was assessed using ATEC, which employs cognitively demanding physical tasks, like an exercise video, to measure executive functions (EFs), memory, and other cognitive processes “in action”. **Results**: Embodied delayed recall was the most frequent impairment (84%), and EF impairments were also common. Among the EF domains, self-regulation was the most frequently impaired at 43%. Using the ATEC total score, 43% of the sample were rated as having a mild or greater level of overall impairment. Strong support for concurrent validity was found for ATEC EF and memory domains when correlated with neurocognitive assessments conceptually related to them. Significant categorical agreement (impaired/not impaired) was also found between neurocognitive testing and ATEC total score. Using the ATEC total score, younger age, higher education, and better premorbid IQ were found to be potential protective factors against cognitive decline. **Conclusions**: Findings support ATEC’s potential for future studies related to AUD and other disorders that may lead to cognitive decline. Embodied cognition may provide new insights into how AUD affects cognition and functioning and be useful to determine what interventions may improve recovery.

## 1. Introduction

Embodied cognition [1], an emerging concept in cognitive neuroscience, proposes that cognitive processes are influenced by bodily experiences and interactions with the environment. Coined in the late 20th century, the term emphasizes the integral role of perception, action, and bodily experiences in shaping human thought and understanding. It represents a departure from the traditional computational models of cognition, offering a holistic perspective on how we perceive and interact with the world. The Automated Test of Embodied Cognition (ATEC) uses cognitively demanding physical tasks to capture cognition in action. It measures balance, motor speed, rhythm, delayed memory, working memory, response inhibition, and self-regulation. ATEC has high internal consistency, excellent test–retest reliability, and strong discriminant and concurrent validity and is a better predictor of function than conventional neurocognitive measures [2].

Alcohol use disorder (AUD) is associated with a progressive decline in cognitive function [3]. According to some studies, up to 80% of patients with AUD may have mild-to-severe cognitive impairment [4,5,6]. These impairments interfere with the acquisition of new learning (e.g., memory) and with better decision making (e.g., executive functioning (EF)), making recovery more difficult. Alcohol-related brain defects and alcohol-associated cognitive impairments may contribute to the progression of AUD by affecting the individual’s ability to benefit from treatment [7] and by impairing their daily community functioning, which in turn increases stress and subsequent relapse [8,9]. In a large study [10], with a mixed sample of substance use disorder (SUD) patients with a mean age of 40 years (SD = 13.9) (N = 656 patients; 391 used alcohol, 123 used cannabis, 100 used stimulants, 26 used opioids), the prevalence of cognitive impairments was 31%. Patients using alcohol had a lower total and memory domain score on the Montreal Cognitive Assessment (MoCA) [11] than those using cannabis. They also reported an association between older age and greater impairment with the greatest association being for the AUD sample (r = −0.33, *p* < 0.01). Not surprisingly, younger patients scored higher than older patients across all SUDs, which may suggest that greater continuing exposure to the effects of substances over time leads to greater cognitive impairment over time.

Others have suggested that education may represent cognitive reserve and serve as a protective factor from acquiring AUD-related neurocognitive impairment. In a study [12] that examined protective factors against cognitive decline with 58 abstaining AUD participants, the investigators reported that 75.9% had some type of cognitive impairment, and that cognitive reserve, defined by education level, served as a protective factor against cognitive impairment.

A systematic review of the published literature on AUD and executive dysfunction [13] found nine studies with neuropsychological assessments and brain imaging and concluded that AUD causes impairments in several cognitive function domains. The review linked those findings to frontal lobe damage. Across the studies, they found that AUD had acute effects on various EF functions, such as updating, set-shifting, and response inhibition, and that some EFs, such as verbal and auditory working memory, are influenced by alcohol more consistently than others. They concluded that a faulty interaction among large-scale networks underlies patients’ executive dysfunction in AUD, which is suggested by changes in prefrontal white-matter pathways.

A review of cognitive impairments in SUD [14] concluded that “altered cognitive function can be viewed as a hallmark feature of substance use disorders and AUD in particular, with documented alterations in the well-known ‘executive’ domains of attention, inhibition/regulation, working memory, and decision-making” (page 102). The authors added that such deficits regulate downstream motivational processes and are fundamental impairments in addiction.

Prior to ATEC, there had not been any comprehensive measurement system of embodied cognition, so there has been no report on how embodied executive functioning or memory may be related to AUD. Here, we offer preliminary insights from ATEC data gathered on a sample with AUD who also received conventional neurocognitive assessments. Analyses offer the first evaluation of embodied cognition in an AUD sample and provide rates of impairment in EFs, memory, and motor speed; examine the relationships between ATEC and conventional cognitive assessments in the context of AUD; and explore the impact of younger age, education, and premorbid IQ as protective factors.

In this study, we hypothesize that ATEC total scores and its domain scores will reveal significant impairments in our AUD sample, that these impairments will be related to age, education, and premorbid IQ, and that ATEC domain scores will be significantly related to the same constructs as measured by standard neurocognitive testing. We will also determine the degree of classification agreement for cognitive impairment between those so categorized by the ATEC total score and those so categorized by neurocognitive test results.

## 2. Materials and Methods

### 2.1. Participants

We are currently conducting a National Institute on Alcohol Abuse and Alcoholism (NIAAA) funded study (RO1AA029075) [15] of cognitive training and donepezil in alcohol use disorder (AUD), for which ATEC is one of the cognitive assessments, and another study on ATEC itself that includes a number of participants with AUD. Because of different protocols, the sample sizes for correlations vary according to whether the measure was used in one protocol or more than one protocol. Inclusion/exclusion criteria for both studies was the presence of a current diagnosis of AUD, as determined using the Mini International Neuropsychiatric Interview (M.I.N.I) [16] and the absence of a psychotic, neurological, sensory, or orthopedic disorder that would interfere with the performance of ATEC. The sample (N = 49) was 69.4% male, with a mean age of 52.06 (14.36), 32.7% were US Veterans, 57.1% had a college degree or higher (mean years of education = 14.63 (2.93)), and 95.9% were right-handed. For those in the NIAAA study (N= 35), the mean of lifetime use of alcohol to intoxication was 247.86 months (194.14), and their Wechsler Test of Adult Reading (WTAR) mean score [17], used as an estimate of premorbid IQ, was 111.00 (12.68).

### 2.2. Measures: Automated Test of Embodied Cognition

ATEC consists of complex tasks related to executive function in motion (e.g., “Cross Your Body” task described below) and a number of simpler movement tasks (e.g., Timed-up and Go, tandem walk, hand pronation–supination, finger tapping) commonly used in neurological exams and utilized in the Movement Disorder Society—Unified Parkinson’s Disease Rating Scale (MDS-UPDRS) [18]. Video administration automates the tasks and increases the fidelity of assessment, while motion capture and artificial intelligence (AI)/machine learning (ML) scoring algorithms decrease measurement error. The system includes a software interface, which is linked to the demonstration video and the motion capture system.

#### 2.2.1. ATEC Novel Tasks

To test higher cognitive processes related to executive functions, novel physical tasks were added to the standard neurological tasks. One of the three core tasks with higher cognitive demand is an attention and response inhibition task called “red light/green light/yellow light (RL/GL/YL)”. It is similar to computerized continuous performance tests (CPT; e.g., [19]) that assess sustained attention and response inhibition but is more complex and requires rhythmic upper body movement in response to commands. The participant is asked to pass a juggling ball from one hand to the other in rhythm to the words “green light”, to move the ball up and down to the words “yellow light”, and to not pass the ball when the participant hears “red light”. The task is subsequently repeated at a faster pace. The participant is then presented with the same task but using a sequence of pictures of green, red, and yellow traffic lights as visual cues, rather than the spoken cues, thus allowing for comparison between sensory modalities. The task is scored for accuracy, response inhibition (not moving on red light), and rhythm (dropping the ball into the opposite hand on the beat).

The second novel task is the “Cross Your Body” task. In beat to a tune, participants are instructed to touch their ears alternately with the opposite hand (left hand to right ear; right hand to left ear) three times and then knees three times (lyrics: “cross your body, touch your ears, ears, ears; cross your body, touch your knees, knees, knees”). Then, participants are instructed to touch knees when the word “ears” is heard and touch ears when “knee” is heard. The same procedure then replaces ears and knees with hips and shoulders. In the final round, all four commands are given, and the person must remember to touch knees when “ears” is heard, ears when “knees” is heard, hips when “shoulders” is heard, and shoulders when “hips” is heard. This task requires sustained attention, working memory, response inhibition, cognitive flexibility, and self-regulation. Crossing the midline increases sensitivity for the detection of subtle brain compromise and increases cognitive load. The task is scored for accuracy and rhythm (touching the body part on the beat).

The third task is an embodied memory task, called “Map Sense”. It requires the participant to remember 3-, 4-, and then, 5-step movements across a 3 × 3 grid. The steps are sequentially displayed on a map on screen, and then, the participant must remember the sequence and move across the grid on the floor in rhythm to a simple tune. There are three trials for each map. The participant is tested again 20 min later without the maps being shown to measure delayed recall. The task is scored for accuracy and rhythm (stepping into the squares on the beat). A list of the ATEC tasks is shown in Table 1, along with the cognitive demands they represent. Expert scoring creates raw scores at the item level (e.g., number of finger taps, accurate ball passes), and these raw scores are then categorized, summed, and converted to a 6-point scale (0–5), so that each component score of each task (e.g., accuracy, rhythm) has equal values. These are added together into 8 domain scores (ATEC internal consistency for the 8 domain scores was excellent at Cronbach’s alpha = 0.904), converted again into a 6-point scale (no impairment to severe impairment; see Scoring Pamphlet in the Supplementary Materials (File S1)). The domain scores are then added together to give the ATEC total score (0–40 points). The ATEC total score is used as a continuous variable and also produces categorical scores for level of impairment from no impairment to very severe impairment. No participants in this study had a score in the very severely impaired range, but all other categories are represented (see Table 2). For this report, data were based on expert scoring (interrater agreement, ICC r = 0.935, *p* < 0.001) [2], because the AI/ML algorithms are still in development. The expert scoring has served as the “ground truth” for the algorithm development. The Scoring Pamphlet is provided in the Supplementary Materials.

#### 2.2.2. Neurocognitive Tests

Attention was evaluated using the Integrated Visual and Auditory (IVA)-2 Continuous Performance Test [20]. Executive function (EF) was measured using the Neuropsychological Assessment Battery (NAB) Mazes [21] and the Wisconsin Card Sorting Test (WCST) [22]. Visual memory was measured using the Brief Visual Memory Test (BVMT) [23] and BVMT delayed recall. Verbal learning and memory was assessed using Hopkins Verbal Learning Test (HVLT) [24] with delayed recall, and story memory was evaluated using Logical Memory I [25] with delayed recall (Logical Memory II).

### 2.3. Procedures

Participants were administered ATEC and other measures, including neurocognitive testing, on the same test day. Procedures were in accord with approved protocols by the IRB at VA Connecticut Healthcare System (VACHS) and included US Veteran and non-Veteran samples. All participants signed a written informed consent document and a HIPAA authorization. All testing was performed in private offices and in the ATEC Lab, a dedicated space for ATEC evaluations that is large enough for a participant to complete the timed up and go task, involving standing up from a seated position and walking 10 feet to a floor marker and walking back. It takes approximately 45 min to complete the ATEC tasks and approximately an hour and a half for neurocognitive assessments. Participants were invited to take breaks as necessary to minimize fatigue.

### 2.4. Analysis

All analyses were performed using SPSS v. 29 0.2.0. ATEC domain and total scores were categorized according to the scoring pamphlet from no impairment to severe impairment (see Supplementary Materials). The cut-offs for the categories were based on clinical practice and the cut-offs for MDS-UPDRS, which was a model for ATEC. A normative sample is not available at this time. Correlations were performed using Pearson’s correlation for parametric data and Spearman’s for non-parametric data. All tests were two-tailed with an alpha of 0.05 for significance. Categorical analyses were performed using Chi-square and intra-class R for inter-rater reliability.

## 3. Results

Categories scoring for each domain revealed that embodied delayed recall had the highest frequency of mild or greater impairment at 84%, and motor speed had the lowest frequency at 2%. Categorized ATEC total scores revealed that 43% of the AUD sample had a mild impairment or greater, and no participants were in the severe category (Table 2).

As predicted, ATEC total scores were significantly correlated with age (N = 49; r = −0.627, *p* < 0.001), indicating that lower ATEC total scores were associated with older age. Age was also associated with more months of life-time alcohol use (N = 35; r = 0.557, *p* < 0.001). ATEC total scores were significantly correlated with education (N = 49; r = 0.504, *p* < 0.001), indicating that more education was associated with better ATEC total scores. ATEC Total scores were also significantly correlated with WTAR total scores (N = 35; r = 0.562, *p* < 0.001), which is generally regarded as a measure of premorbid IQ.

To manage issues related to multiple comparisons, we began by correlating the ATEC total scores with two key EF assessments and three key memory assessments. ATEC total scores were strongly correlated with EF measures’ Mazes total score (r = 0.501, *n* = 49, *p* < 0.001, Cohen’s d’ = 1.15) and WCST categories correct (r = 0.643, *n* = 34, *p* < 0.001, Cohen’s d’ = 1.66). ATEC total scores were highly correlated with BVMT total scores (r = 0.743, *n* = 37, *p* < 0.001, Cohen’s d’ = 2.25) and with Logical Memory I (r = 0.714, *n* = 35, *p* < 0.001, Cohen’s d’ = 2.01. It was not significantly correlated with HVLT (r = 0.292, *n* = 35, *p* < 0.088, Cohen’s d’ = 0.62); although, the effect size was moderate. The Bonferroni correction for multiple comparisons did not change the significance of these findings.

Having established the strength of the correlation between the ATEC total score and EF and memory measures, we then explored the correlations of the individual ATEC domains with a number of EF and memory variables. We did not correct for multiple comparisons in this exploratory analysis.

Correlations between ATEC EF domains and the neurocognitive tests for EF revealed strong relationships (Table 3). ATEC balance was included with the EF domains, because exploratory factor analyses have found that it loads on the same factor with the other EF domains. It showed a significant relationship with Mazes and with IVA scores but not with WCST. ATEC attention was very strongly related to IVA scores and also with Mazes, though, like ATEC balance, not with WCST. The other ATEC EF domains showed strong correlations with Mazes and WCST but not with IVA.

The correlations between ATEC memory domains (Table 4) show significant relationships between ATEC working memory and HVLT delayed recall, logical memory, and digit span. ATEC embodied delayed recall showed significant relationships with BVMT total, BVMT delayed recall Logical Memory I, Logical Memory II, and digit span.

Finally, participants were categorized as impaired or not impaired using neurocognitive testing and ATEC total scores. Participants who had two or more neurocognitive test scores 1.5 standard deviations (SD) below the age-corrected norms were categorized as impaired, and that classification was cross-tabulated with the ATEC total scores of mild or greater impairment in a 2X2 Chi-square analysis. Of 35 AUD participants with neurocognitive testing, 11 (31.4%) met the criteria for being impaired, and 11 were also identified as impaired on the ATEC total score. Eight of the eleven (72.7%) were the same individuals (Chi-sq = 12.696, df = 1, *p* < 0.001). Intraclass correlation was also significant (intraclass R = 0.752, F = 4.029, df = 34, *p* < 0.001).

## 4. Discussion

This is the first report on the assessment of embodied cognition in an AUD sample. Using the ATEC total score, 21 out of 49 (43%) were classified as having mild or greater overall impairment, a percentage in keeping with much of the literature cited above. The most frequent and severe impairments were in embodied delayed recall with 41 out of 49 (84%) having mild or greater impairment. Motor speed was not found to be a problem in this sample, with only 1 participant having mild impairment, but balance impairments were more common, with 10 out of 49 (20.40%) showing a mild or moderate impairment. Among the EF domains (working memory, response inhibition, self-regulation, rhythm, and attention), self-regulation impairments were the most common and severe, with 21 out of 49 (43%) with scores from mild to severe. Given the role that self-regulation plays in addiction and recovery [26,27], this is a meaningful finding, and one that would suggest future studies to determine its predictive value for rehabilitation outcomes. Attention was not found to be a problem in this sample, with only one participant having a mild impairment.

A review article [28] on alcohol-related dementia (ARD) found 72 articles that supported the strong association between AUD and cognitive impairments, concluding that chronic alcohol use affects visuospatial functions, memory, and executive tasks, with a potential for partial recovery if abstinence is maintained. Our ATEC findings support that conclusion, with 43% having mild or greater overall impairment with the highest frequency of impairment in embodied memory recall and self-regulation.

For the participants who had neurocognitive assessments, we found strong support for the construct validity of the ATEC total score and for the ATEC measures of EF and memory. ATEC working memory, response inhibition, and self-regulation had significant correlations with both Mazes and WCST, which strongly supports their being measures of EF. Balance and rhythm, though not usually associated with EF, were included in this comparison with EF cognitive tests because of a small amount of literature that has found such associations [29,30,31]. ATEC balance was correlated significantly with Mazes and with IVA-2, providing modest support for a relationship with EF, and rhythm and coordination were related to both Mazes and WCST. These findings suggest that future investigation is warranted on these important embodied cognition domains and EFs. ATEC attention was most strongly associated with the computerized IVA continuous performance test supporting the construct validity of the ATEC attention domain. IVA-2 provides sub-scores for auditory attention (r = 0.418, *p* < 0.05) and visual attention (r = 0.600, *p* < 0.001), and it appears that ATEC attention may be somewhat more aligned with visual attention, though the difference between the two correlations is not significant (Z-score = −0.99, *p* = 0.32).

Construct validity was also found for ATEC working memory and embodied delayed recall. HVLT list learning immediate memory was not associated with the ATEC memory domains, but HVLT delayed recall was related to ATEC working memory though not embodied delayed recall. Visual memory on the BVMT was much more strongly related to our ATEC memory scores. A comparison between ATEC working memory’s correlation with HVLT total recall (r = 0.281) and BVMT total recall (r = 0.652) is significant (Z-score = −2.01, *p* = 0.045). Given the modest sample size, this finding does suggest that ATEC may more closely relate to visual recall. Yet, both ATEC memory domains have significant correlations with story memory, particularly for Logical Memory I. So, it may be that the narrative sequence of story memory and remembering movements in sequence are making similar cognitive demands. These findings may encourage further explorations of these embodied cognitive processes, as they relate to sequence.

It is interesting that ATEC total scores had a robust relationship with EF and memory measures with very large effect sizes, greater than the relationship of any of the specific ATEC domains from which it is derived. The implication is that embodied cognition is really a unifying construct for cognition in action with powerful explanatory potential beyond that of any single measure. As we learn more about ATEC in a range of clinical samples, we will learn whether this is a generalizable finding that supports the significance of ATEC for understanding EF, memory, and other cognitive functions, as dependent on the mind and body working together.

We also examined the classification agreement between our neurocognitive tests and ATEC total scores and found significant and meaningful agreement. If we were to regard the neurocognitive tests cut-off (two tests 1.5 SD below the age corrected norm) as “gold standard”, then we could conclude that the false-positive rate (12.5%) and the false-negative rate (12.5%) are equal, so that no clear bias was detected. Since most participants were correctly classified, it appears that ATEC total scores have a concordance with neurocognitive testing overall.

Finally, our findings with ATEC support the argument that younger age, higher education, and better premorbid IQ are protective factors against cognitive decline in this AUD sample. Education is a complicated variable, because it may also reflect socio-economic status and the income of parents, but the significant relationship with premorbid IQ would seem to lend support to the interpretation that cognitive reserve is involved.

### Limitations

The sample size is relatively small and may not fully represent the range of patients who have AUD. ATEC is not yet a normed instrument, and the cut-offs for the severity of impairment are based on that of the MDS-UPDRS and clinical practice. Nevertheless, correlations with normed neurocognitive testing produced confirmatory evidence for the construct validity of the ATEC total scores and for the specific ATEC domain scores. Finally, it is important to note that significant correlations still leave a great deal of unexplained variance. Further investigation will be needed to learn more about what ATEC is capturing that is distinct from standard neurocognitive tests.

## 5. Conclusions

ATEC is a new instrument. Its high-fidelity administration and breadth of assessment offers a new tool to investigate the link between AUD and neurocognitive decline. By integrating body and mind through cognitively demanding physical tasks, ATEC may yield findings that will be more sensitive to the true nature of the impairments associated with AUD and other substance use disorders. These findings support the construct validity of ATEC and its potential role in future AUD and SUD research. Embodied cognition may provide new insights into how AUD affects cognition and functioning and be useful in studies about interventions that may improve cognitive recovery in people with AUD. ATEC may be particularly sensitive to detecting cognitive improvement from rehabilitation strategies that include cognitively demanding physical activity, such as appropriate sports, or rhythmic training, such as dance therapy. ATEC may offer clinicians and researchers a new tool in the ongoing effort to improve the lives of individuals with AUD.

## 6. Patents

ATEC is the intellectual property of the VA, Yale University, and the University of Texas at Arlington. Internation and national patent protection is in process.

## Figures and Tables

**Table 1:** ATEC tasks, cognitive demands, and scoring categories.

Type	Tasks	Domains
Gross Motor and Gait, Proprioception, Vestibular ^1^	Timed Up and Go, Dual Attention, Tandem Gait, Romberg, and Standing on One Foot (L, R)	Balance and Gait
Rhythmic Movement ^2^	Marching Slow and Fast	Rhythm
Embodied Memory ^2^	3, 4, 5 Step Maps	Working Memory/Rhythm
Embodied Delayed Recall ^2^	20 min Delayed Recall	Delayed Recall
Bilateral Coordination ^1^	Ball Pass to the Beat Slow and Fast	Coordination/Rhythm
Response Inhibition ^2^	Red Light/Yellow Light/Green Light Slow and Fast/Auditory and Visual	Response Inhibition, Rhythm, Attention
Bilateral Coordination and Self-Regulation ^2^	Cross Your Body	Self-Regulation, Rhythm, and Working Memory
Rapid Sequential Movements ^1^	Foot Tap, Foot Stomp, Fist Open and Close, Hand Pronate/Supinate, and Finger Tap (all L, R)	Motor Speed and Fluidity

^1^ MDS-UPDRS; ^2^ higher cognitive function.

**Table 2:** Frequency of impairment (N = 49) by cognitive domain and ATEC total score.

Domain	Not Impaired	Very Mild	Mild	Moderate	Severe
Balance	25 (51%)	14 (28%)	8 (16.3%)	2 (4.1%)	0
Working Memory	31 (63.3)	15 (30.6)	3 (6.1%)	0	0
Response Inhibition	20 (40.8%)	16 (32.7%)	11 (22.4%)	2 (4.1%)	0
Self-Regulation	22 (44.9%)	6 (12.2%)	17 (34.7%)	3 (6.1%)	1 (2.0%)
Rhythm and Coordination	25 (51%)	15 (30.6%)	6 (12.2%)	2 (4.1%)	1 (2.0%)
Attention	39 (79.6%)	9 (18.4%)	1 (2.0)	0	0
Embodied Memory Delayed Recall	5 (10.2%)	3 (6.1%)	19 (38.8%)	11 (22.4%)	11 (22.4%)
Motor Speed	42 (85.7%)	6 (12.2%)	1 (2.0%)	0	0
ATEC Total Score	17 (34.7%)	11 (22.4%)	15 (30.6%)	5 (10.2%)	1 (2.0%)

**Table 3:** ATEC EF domains and EF neurocognitive tests.

EF Neurocognitive Tests	ATEC EF Domains					
	Balance	Working Memory	Response Inhibition	Self-Regulation	Rhythm and Coordination	Attention
Mazes ^1^	0.376 **	0.389 **	0.478 ***	0.495 ***	0.347 *	0.395 **
WCST ConceptualResponses ^2^	0.264	0.444 **	0.428 **	0.358 *	0.454 **	0.246
IVA Full Scale ^3^	0.352 *	0.315	0.146	0.136	−0.170	0.524 **
IVA Auditory ^3^	0.345 *	0.194	0.067	0.054	−0.206	0.418 *
IVA Visual ^3^	0.339	0.442 **	0.273	0.275	−0.036	0.600 ***

* *p* < 0.05, ** *p* < 0.01, *** *p* < 0.001; ^1^ *n* = 49; ^2^ *n* = 34; ^3^ *n* = 35.

**Table 4:** ATEC memory domains and verbal and visual memory neurocognitive tests.

Neurocognitive Tests of Memory	Working Memory	Embodied Delayed Recall
^1^ HVLT Total Recall	0.281	0.296
^2^ HVLT Delayed Recall	0.487 **	0.310
^1^ Digit Span Total	0.393 *	0.390 *
^3^ BVMT Total Recall	0.652 ***	0.468 **
^3^ BVMT Delayed Recall	0.659 ***	0.511 **
^1^ Logical Memory I	0.557 **	0.589 **
^2^ Logical Memory II	0.337 *	0.424 *

* *p* < 0.05, ** *p* < 0.01, *** *p* < 0.001; ^1^ *n* = 35; ^2^ *n* = 34; ^3^ *n* = 37.

## Data Availability

Data collection is ongoing and has not been archived yet. The data presented in this manuscript are available by contacting the corresponding author.

## Supplementary Materials

The following supporting information can be downloaded at: https://www.mdpi.com/article/10.3390/brainsci15030228/s1, File S1: ATEC scoring pamphlet.

## Abbreviations

The following abbreviations are used in this manuscript:

ATEC	Automated Test of Embodied Cognition
AUD	Alcohol use disorder
EF	Executive function
SUD	Substance use disorder
MoCA	Montreal Cognitive Assessment
NIAAA	National Institute on Alcohol Abuse and Alcoholism
M.I.N.I.	Mini International Neuropsychiatric Interview
WTAR	Wechsler Test of Adult Reading
MDS-UPDRS	Movement Disorder Society—Unified Parkinson’s Disease Rating Scale
AI	Artificial intelligence
ML	Machine learning
IVA	Integrated Visual and Auditory-2 Continuous Performance test
NAB	Neuropsychological Assessment Battery
WCST	Wisconsin Card Sorting Test
BVMT	Brief Visual Memory Test
HVLT	Hopkins Verbal Learning Test
VACHS	VA Connecticut Healthcare System

## References

[1] Wilson A.D., Golonka S. (2013). Embodied cognition is not what you think it is. Front. Psychol..

[2] Bell M.D., Hauser A.J., Weinstein A.J. (2023). The Automated Test of Embodied Cognition: Concept, Development, and Preliminary Findings. Brain Sci..

[3] Bernardin F., Maheut-Bosser A., Paille F. (2014). Cognitive impairments in alcohol-dependent subjects. Front. Psychiatry.

[4] Bates M.E., Bowden S.C., Barry D. (2002). Neurocognitive impairment associated with alcohol use disorders: Implications for treatment. Exp. Clin. Psychopharmacol..

[5] Meek P.S., Clark H.W., Solana V.L. (1989). Neurocognitive impairment: The unrecognized component of dual diagnosis in substance abuse treatment. J. Psychoact. Drugs.

[6] Sullivan E.V., Fama R., Rosenbloom M.J., Pfefferbaum A. (2002). A profile of neuropsychological deficits in alcoholic women. Neuropsychology.

[7] Blume A.W., Schmaling K.B., Marlatt G.A. (2005). Memory, executive cognitive function, and readiness to change drinking behavior. Addict. Behav..

[8] Blume A.W., Alan Marlatt G. (2009). The role of executive cognitive functions in changing substance use: What we know and what we need to know. Ann. Behav. Med..

[9] Bowden S.C., Crews F.T., Bates M.E., Fals-Stewart W., Ambrose M.L. (2001). Neurotoxicity and neurocognitive impairments with alcohol and drug-use disorders: Potential roles in addiction and recovery. Alcohol. Clin. Exp. Res..

[10] Bruijnen C.J., Dijkstra B.A., Walvoort S.J., Markus W., VanDerNagel J.E., Kessels R.P., De Jong C.A. (2019). Prevalence of cognitive impairment in patients with substance use disorder. Drug Alcohol Rev..

[11] Nasreddine Z.S., Phillips N.A., Bedirian V., Charbonneau S., Whitehead V., Collin I., Cummings J.L., Chertkow H. (2005). The Montreal Cognitive Assessment, MoCA: A brief screening tool for mild cognitive impairment. J. Am. Geriatr. Soc..

[12] Requena-Ocaña N., Araos P., Flores M., García-Marchena N., Silva-Peña D., Aranda J., Rivera P., Ruiz J.J., Serrano A., Pavón F.J. (2021). Evaluation of neurotrophic factors and education level as predictors of cognitive decline in alcohol use disorder. Sci. Rep..

[13] Maharjan S., Amjad Z., Abaza A., Vasavada A.M., Sadhu A., Valencia C., Fatima H., Nwankwo I., Anam M., Mohammed L. (2022). Executive dysfunction in patients with alcohol use disorder: A systematic review. Cureus.

[14] Ramey T., Regier P.S. (2019). Cognitive impairment in substance use disorders. CNS Spectr..

[15] Yoon G., Sofuoglu M., Petrakis I.L., Pittman B., Bell M.D. (2024). The combination of donepezil and cognitive training for improving treatment outcomes for alcohol use disorder: Design of a randomized controlled trial. Contemp. Clin. Trials.

[16] Sheehan D.V., Lecrubier Y., Sheehan K.H., Amorim P., Janavs J., Weiller E., Hergueta T., Baker R., Dunbar G.C. (1998). The Mini-International Neuropsychiatric Interview (MINI): The development and validation of a structured diagnostic psychiatric interview for DSM-IV and ICD-10. J. Clin. Psychiatry.

[17] Wechsler D. (2001). Wechsler Test of Adult Reading: WTAR.

[18] Goetz C.G. (2010). Unified Parkinson’s Disease Rating Scale (UPDRS) and The Movement-Disorder Society Sponsored-unified Parkinson’s Disease Rating Scale (MDS-UPDRS). Encycl. Mov. Disord..

[19] Conners C.K., Staff M. (2000). Conners’ Continuous Performance Test II: Computer Program for Windows Technical Guide and Software Manual.

[20] Sanford J., Turner A. (2002). Integrated Visual and Auditory Continuous Performance Test Manual.

[21] Stern R.A., White T. (2003). NAB, Neuropsychological Assessment Battery: Administration, Scoring, and Interpretation Manual.

[22] Heaton R.K., Chelune G.J., Talley J.L., Kay G.G., Curtiss G. (1993). Wisconsin Card Sorting Test Manual: Revised and Expanded.

[23] Benedict R. (1997). Brief Visuospatial Memory Test-Revised Professional Manual.

[24] Brandt J. (1991). The Hopkins Verbal Learning Test: Development of a new memory test with six equivalent forms. Clin. Neuropsychol..

[25] Wechsler D. (1987). Wechsler Memory Scale Revised.

[26] Baumeister R.F., Vonasch A.J. (2015). Uses of self-regulation to facilitate and restrain addictive behavior. Addict. Behav..

[27] Sayette M.A., Griffin K.M. (2004). Self-regulatory failure and addiction. Handb. Self-Regul. Res. Theory Appl..

[28] Sachdeva A., Chandra M., Choudhary M., Dayal P., Anand K.S. (2016). Alcohol-Related Dementia and Neurocognitive Impairment: A Review Study. Int. J. High Risk Behav. Addict..

[29] Iersel M.B.v., Kessels R.P., Bloem B.R., Verbeek A.L., Olde Rikkert M.G. (2008). Executive functions are associated with gait and balance in community-living elderly people. J. Gerontol. Ser. A Biol. Sci. Med. Sci..

[30] Wang Y.N., Wen X.N., Chen Y., Xu N., Zhang J.H., Hou X., Liu J.P., Li P., Chen J.Y., Wang J.H. (2024). Effects of movement training based on rhythmic auditory stimulation in cognitive impairment: A meta-analysis of randomized controlled clinical trial. Front. Neurosci..

[31] Vazou S., Klesel B., Lakes K.D., Smiley A. (2020). Rhythmic physical activity intervention: Exploring feasibility and effectiveness in improving motor and executive function skills in children. Front. Psychol..

