# Supplementary material for: Embodied Cognition and Alcohol Use Disorder: Frequency of Impairments and Relationship to Neurocognitive Assessments
Source: Brain Sci. 2025 Feb 22;15(3):228. doi: 10.3390/brainsci15030228 (PMC11940586; doi:10.3390/brainsci15030228)
Supplement: Supplementary file 1 [file brainsci-15-00228-s001.zip › brainsci-3453170-supplementary.pdf]

Rater:_______________________________________ Date:________________ Subject ID:_____________________________

AUTOMATED TEST of EMBODIED COGNITION (ATEC)

Morris D. Bell, Ph.D.

A. GROSS MOTOR—GAIT AND BALANCE

	RAW	CONVERTED	***SCORING CRITERIA***
1. Timed-Up & Go			Steps > 20 = 0, 20-16=1, <16 = 2
2. Dual Attention			Steps > 20 = 0, 20-16=1, <16 = 2
3. Tandem Gait Forward			Steps correct: <4=0, 4-6 = 1, 7-8 = 2
4. Stand - Arms Outstretched			Seconds: <5 = 0, 5 - 8 = 1, 9 - 10 = 2
5. Stand on One Foot—Right			Seconds: <5 = 0, 5 - 8 = 1, 9 - 10 = 2
6. Stand on One Foot—Left			Seconds: <5 = 0, 5 - 8 = 1, 9 - 10 = 2
A.7. Counting Backwards 93,86,79,72,65			Numbers Correct 0=0, 1=1, 2=2, 3=>3

A.1 TOTAL GAIT/BALANCE SCORE (ITEM 1 + ITEM2 + ITEM 3)

SCORE 0 - 6 ☐

A.2 TOTAL BALANCE SCORE (ITEM 4 + ITEM 5 + ITEM 6)

SCORE 0 - 6 ☐

B. RHYTHMIC MOVEMENT

**Raw Score = total number of steps in rhythm (step on the beat)*

	RAW	CONVERTED	
1. March to the Beat—Slow			Beats correct: 0-8= 0, 9-16 = 1, 17-20 = 2
2. March to the Beat—Fast			Beats correct: 0-16 = 0, 17-28= 1, 29-32 = 2

B.1 TOTAL RHYTHMIC MOVEMENT SCORE

SCORE 0– 4 ☐

Rater:______________________ Date:____________ Subject ID:________________

C. Visuospatial Memory

		ACCURACY RAW	ACCURACY CONVERTED
1. MapSense3 Trial 1	0-1=0, 2=1, 3=2		
2. MapSense3 Trial 2	0-1=0, 2=1, 3=2		
3. MapSense3 Trial 3	0-1=0, 2=1, 3=2		
4. MapSense4 Trial 1	0-1=0, 2-3=1, 4=2		
5. MapSense4 Trial 2	0-1=0, 2-3=1, 4=2		
6. MapSense4 Trial 3	0-1=0, 2-3=1, 4=2		
7. MapSense5 Trial 1	0-2=0, 3-4=1, 5=2		
8. MapSense5 Trial 2	0-2=0, 3-4=1, 5=2		
9. MapSense5 Trial 3	0-2=0, 3-4=1, 5=2		

RHYTHM/COORDINATION

		1
3	2	

	RAW	CONVERTED
0-1=0, 2=1, 3=2		
0-1=0, 2=1, 3=2		

1		
	2	
	3	4

	RAW	CONVERTED
0-1=0, 2-3=1, 4=2		
0-1=0, 2-3=1, 4=2		

	1	
	2	3
5	4	

	RAW	CONVERTED
0-2=0, 3-4=1, 5=2		
**0-2=0, 3-4=1, 5=2**		

C.1 TOTAL VISUOSPATIAL MEMORY ACCURACY SCORE

SCORE 0 - 18 ☐

C.2 TOTAL VISUOSPATIAL MEMORY RHYTHM/ COORDINATION SCORE

SCORE 0 - 12 ☐

D. BILATERAL COORDINATION

**each pass get up two rhythm points; one for each beat*

	ACCURACY RAW	ACCURACY CONVERTED	RHYTHM RAW	RHYTHM CONVERTED
1. Bi-manual Ball Pass—Slow				
2. Bi-manual Ball Pass—Fast				

Passes correct: 0 - 4 = 0, 5– 6 = 1, 7– 8 = 2

Rhythm: 0 - 8 = 0, 9– 12 = 1, 13– 16 = 2

D.1 TOTAL BILATERAL COORDINATION

ACCURACY SCORE 0– 4 ☐

D.2 TOTAL BILATERAL COORDINATION

RHYTHM SCORE 0– 4 ☐

Rater:______________________________________ Date:_______________ Subject ID:___________________________

E. AUDITORY UPPER BODY ACCURACY, RHYTHM AND RESPONSE INHIBITION

**0: moves during both beats, 1: moves only on first, 2: still*

Green Lights correct: 0 - 5= 0, 6– 8= 1, 9– 10= 2	Red Lights correct: 0 - 6 = 0, 7-10= 1, 11-12= 2	Green Lights Rhythm: 0 - 10 = 0, 11-16= 1, 17- 20= 2

Bi-manual Ball Pass Red/Green Light	GREEN LIGHTS CORRECT		RED LIGHTS CORRECT		GREEN RHYTHM CORRECT	
	RAW	CONVERTED	RAW	CONVERTED	RAW	CONVERTED
1. Auditory—Slow	☐	☐	☐	☐	☐	☐
2. Auditory —Fast	☐	☐	☐	☐	☐	☐

Green/Yellow Lights correct: 0 - 6= 0, 7– 10= 1, 11– 12= 2	Red Lights correct: 0 - 4 = 0, 5-6= 1, 7-8= 2	Green/Yellow Lights Rhythm: 0 - 12 = 0, 13-20= 1, 21-24= 2

Ball Pass Red/Green/Yellow Light	GREEN/YELLOW LIGHTS CORRECT		RED LIGHTS CORRECT		G/Y RHYTHM CORRECT	
3. Auditory—Slow	☐	☐	☐	☐	☐	☐
4. Auditory —Fast	☐	☐	☐	☐	☐	☐

E.1 TOTAL AUDITORY ACCURACY

SCORE 0 - 8 ☐

E.2 TOTAL AUDITORY RESPONSE INHIBITION

SCORE 0 - 8 ☐

E.3 TOTAL AUDITORY RHYTHM

SCORE 0 - 8 ☐

E.4 TOTAL G/Y LIGHT ACCURACY

SCORE 0 - 4 ☐

E.5 TOTAL RED LIGHT ACCURACY

SCORE 0 - 4 ☐

F. VISUAL UPPER BODY ACCURACY, RHYTHM AND RESPONSE INHIBITION

*** No beats (rhythm)*

0: movement during the whole video

1: self-correct (move and then keep still)

2: no movement — still

Green/Yellow Lights correct: 0 - 6= 0, 7– 10= 1, 11– 12= 2	Red Lights correct: 0 - 4 = 0, 5-6= 1, 7-8= 2

Bag Pass Red/Green/Yellow Light	GREEN/YELLOW LIGHTS CORRECT		RED LIGHTS CORRECT	
1. Visual—Slow	☐	☐	☐	☐
2. Visual —Fast	☐	☐	☐	☐

F.1 TOTAL VISUAL ACCURACY

SCORE 0 - 4 ☐

F.2 TOTAL VISUAL RESPONSE INHIBITION

SCORE 0 - 4 ☐

Rater:________________________ Date:____________ Subject ID:____________________

G. BI-LATERAL COORDINATION AND SELF-REGULATION

**Accuracy Raw Score = total number of body parts touched at least once*

**Rhythm/Coordination Raw Score = total number of times correctly touching body part with the opposite hand all three times, accurately keeping the beat for all three touches. Starting late and rushing to catch the beat is not correct*

	Accuracy scale	Accuracy RAW SCORE	Accuracy CONVERTED	Rhythm/Coordination scale	Rhythm/Coordination RAW SCORE	Rhythm/Coordination CONVERTED
1. Trial 1—Ears, Shoulders, Hips and Knees<br>**If Converted Accuracy score = 0, go to H.*	0-1=0, 2-3=1, 4=2			0-6=0, 7-10=1, 11-12=2		
2. Trial 2—Opposite Ears and Knees<br>**If Converted Accuracy score = 0, go to H.*	0-4=0, 5-7=1, 8=2			0-12=0, 13-20=1, 21-24=2		
3. Trial 3—Opposite Hips and Shoulders<br>**If Converted Accuracy score = 0, go to H.*	0-4=0, 5-7=1, 8=2			0-12=0, 13-20=1, 21-24=2		
4. Trial 4—Opposite Ears, Knees, Hips and Shoulders	0-6=0, 7-10=1, 11-12=2			0-18=0, 19-32=1, 33-36=2		

G.1 TOTAL ACCURACY SCORE

SCORE 0 - 8 ☐

G.2 TOTAL RHYTHM/COORDINATION SCORE 0 - 8 ☐

G.3 WORKING MEMORY ACCURACY SCORE (#2,#3,#4)

SCORE 0 - 6 ☐

H. Embodied Memory Recall

	Accuracy scale	ACCURACY RAW	ACCURACY CONVERTED	Grid	Rhythm/Coordination scale	RHYTHM/COORDINATION RAW	RHYTHM/COORDINATION CONVERTED
1. MapSense3 Recall	0-1=0, 2=1, 3=2			_ _ 1 / 3 2 _ / _ _ _	0-1=0, 2=1, 3=2		
2. MapSense4 Recall	0-1=0, 2-3=1, 4=2			1 _ _ / _ 2 _ / _ 3 4	0-1=0, 2-3=1, 4=2		
3. MapSense5 Recall	0-2=0, 3-4=1, 5=2			_ 1 _ / _ 2 3 / 5 4 _	0-2=0, 3-4=1, 5=2		

H.1 TOTAL EMBODIED MEMORY RECALL ACCURACY SCORE

SCORE 0 - 6 ☐

H.2 TOTAL EMBODIED MEMORY RECALL RHYTHM/COORDINATION SCORE

SCORE 0 - 6 ☐

I. RAPID/SEQUENTIAL MOVEMENTS SCORING CRITERIA

1. FOOT TAP -Rapidity Raw score = total number of correct foot taps in 10 seconds.
0-9 =0; 10-19 = 1; >20 = 2

2. FOOT STOMP- Rapidity Raw score = total number of correct foot stomp movements in 10 seconds.
0-9 =0; 10-19 = 1; >20 = 2

3. FIST OPEN CLOSE -Rapidity Raw score = total number of correct fist opening times in 10 seconds.
0-9 =0; 10-19 = 1; >20 = 2

4. HAND PRONATE/SUPINATE -Rapidity Raw score = total number of correct Pronate/Supinate movements in 10 seconds. 0-5 =0; 6-9 = 1; >10 = 2

5. FINGER TAP -Rapidity Raw score = total number of correct Finger taps in 10 seconds.
0-15 =0; 16-29 = 1; >30 = 2

****Fluidity Score ALL TASKS** — Smoothness and accuracy of motion (freezes up or other movements intrude).

Stops and doesn't restart or switches to another movement and doesn't change back to the correct movement = 0.

Stops but restarts, has intrusive movements but returns to correct movements or has mirror movements = 1.

Continuous correct movement = 2.

H. RAPID/SEQUENTIAL MOVEMENTS

FLUIDITY CONVERTED SCORE

RAPIDITY CONVERTED SCORE

RAPIDITY RAW SCORE

FOOT TAP—RIGHT FOOT (10 SEC)				
FOOT TAP—LEFT FOOT (10 SEC)				
FOOT STOMP—RIGHT FOOT (10 SEC)				
FOOT STOMP—LEFT FOOT (10 SEC)				
FIST OPEN CLOSE —RIGHT HAND (10 SEC)				
FIST OPEN CLOSE —LEFT HAND (10 SEC)				
HAND PRONATE/SUPINATE—RIGHT HAND (10 SEC)				
HAND PRONATE/SUPINATE—LEFT HAND (10 SEC)				
FINGER TAP—RIGHT HAND (10 SEC)				
FINGER TAP—LEFT HAND (10 SEC)				
TOTAL SCORE				

I.1 TOTAL RAPIDITY SCORE

I.2 TOTAL FLUIDITY SCORE

Rater:________________________ Date:______________ Subject ID:________________________

SUMMARY OF SCORES

BALANCE

Gait/Balance Total (A.1) ☐ + Balance Total (A.2) ☐ = Balance Total ☐

0-1 = 0; 2-3 = 1, 4-5 = 2; 6-8 = 3; 9-10 = 4; 11-12 = 5

BALANCE CONVERTED SCORE ☐

WORKING MEMORY

Yellow Light Accuracy Total (E.1+F.1) ☐ + Cross Body Oppo-sites (G.3) ☐ + MapSense (C.1) ☐ + Counting Backwards (A.7) ☐ = Working Memory Total ☐

0-9 = 0; 10-16 = 1; 17-24 = 2; 25-30 = 3; 31-35 = 4; 36-39 = 5

WORKING MEMORY CONVERTED SCORE ☐

RESPONSE INHIBITION

Bag Pass Response Inhibition Total (E.2+F.2) ☐ + Cross Body Rhythm Total (G.2) ☐ = Response Inhibition Total ☐

0-3 = 0; 4-6 = 1, 7-10 = 2; 11-14 = 3; 15-17 = 4; 18-20= 5

RESPONSE INHIBITION CONVERTED SCORE ☐

SELF-REGULATION

Cross Body Accuracy (G.1) ☐ + Cross Body Rhythm Total (G.2) ☐ = Self-Regulation Total ☐

0-1 = 0; 2-3 = 1, 4-8 = 2; 9-12 = 3; 13-14 = 4; 15-16 = 5

SELF-REGULATION CONVERTED SCORE ☐

Rhythm/Coordination

Upper Body Rhythm Total (D.2+E.3) ☐ + Whole Body Rhythm Total (G.2) ☐ + MapSense (C.2) ☐ + Embodied Memory Recall Rhythm/ Coordination (H.2) ☐ = Rhythm/Coordination Total ☐

0-5 = 0; 6-12 = 1; 13-18 = 2; 19-25 = 3; 26-32 = 4; 33-38 = 5

RHYTHM/COORDINATION CONVERTED SCORE ☐

Rater:____ Date:________________ Subject ID:_____________________________

ATTENTION

Bag Pass Accuracy Total (D.1+E.1+F.1)		Cross Body Accuracy Total (G.1)		MapSense (C.1)		Attention Total
☐	+	☐	+	☐	=	☐

0-5 = 0; 6-13 = 1; 14-20 = 2; 21-30 = 3; 31-37 = 4; 38-42 = 5

ATTENTION CONVERTED SCORE ☐

RAPIDITY MOTOR SPEED

Rapidity Total (I.1)		Rapidity Motor Speed Total
☐	=	☐

0-3 = 0; 4-6 = 1; 7-10 = 2; 11-15 = 3; 16-18 = 4; 19-20 = 5

RAPIDITY MOTOR SPEED CONVERTED SCORE ☐

EMBODIED MEMORY RECALL

Embodied Memory Recall Accuracy (H.1)		Embodied Memory Recall
☐	=	☐

0= 0; 1= 1; 2 = 2; 3-4 = 3; 5 = 4; 6 = 5

EMBODIED MEMORY RECALL CONVERTED ☐

FLUIDITY MOTOR SPEED

Fluidity Total (I.2) ☐

Fluidity Motor Total

FLUIDITY MOTOR SPEED CONVERTED SCORE ☐

0-3 = 0; 4-6 = 1; 7-10 = 2; 11-15 = 3; 16-18 = 4; 19-20 = 5

Rater:________________________ Date:______________ Subject ID:__________________________

AMC TOTAL SCORE

BALANCE CONVERTED SCORE ☐

+

WORKING MEMORY CONVERTED SCORE ☐

+

RESPONSE INHBITION CONVERTED SCORE ☐

+

SELF-REGULATION CONVERTED SCORE ☐

+

RHYTHM/COORDINATION CONVERTED SCORE ☐

+

ATTENTION CONVERTED SCORE ☐

+

EMBODIED MEMORY RECALL CONVERTED SCORE ☐

+

RAPIDITY MOTOR SPEED CONVERTED (See Notes) ☐

=

AMC TOTAL SCORE ☐

Performance Categories

**Very Severely Impaired**	**0—15**	☐
**Severely Impaired**	**16—18**	☐
**Moderately Severely Impaired**	**19—22**	☐
**Moderately Impaired**	**23—27**	☐
**Mildly Impaired**	**28—32**	☐
**Minor Difficulties**	**33—36**	☐
**No Difficulties**	**37—40**	☐

Note: Motor speed fluidity is no long included in “Motor Speed Converted Score” .
Motor Speed Fluidity Score is shown on page 9 and is a Supplemental Score not included in overall “AMC TOTALE SCORE”.

SUPPLEMENTARY SCORES

Discrepancy - Right / Left

Right	Left
A.5	A.6
H.1	H.2
H.3	H.4
H.5	H.6
H.7	H.8
H.9	H.10
Total =	Total =

☐ − ☐ = ☐

Discrepancy - Auditory / Visual

Auditory	Visual
E.4	F.1
E.5	F.2
Total =	Total =

☐ − ☐ = ☐

Discrepancy

Timed Up & Go / Dual Attention

☐ − ☐ = ☐

Discrepancy - Accuracy / Response Inhibition

Accuracy	Response Inhibition
E.1	E.2
F.1	F.2
Total =	Total =

☐ − ☐ = ☐

Converted Motor Speed Fluidity Score Total

☐
